# Molecular characterization of a novel amplicon at 1q21-q22 frequently observed in human sarcomas.

**DOI:** 10.1038/bjc.1998.521

**Published:** 1998-08

**Authors:** A. Forus, J. M. Berner, L. A. Meza-Zepeda, G. Saeter, D. Mischke, O. Fodstad, O. Myklebost

**Affiliations:** Department of Tumour Biology, The Norwegian Radium Hospital, Oslo.

## Abstract

**Images:**


British Journal of Cancer (1998) 78(4), 495–503
© 1998 Cancer Research Campaign

### Molecular characterization of a novel amplicon at 1q21–q22 frequently observed in human sarcomas

**A Forus[1], J-M Berner[1], LA Meza-Zepeda[1], G Saeter[2], D Mischke[3], Ø Fodstad[1] and O Myklebost[1]**

Departments of [1]Tumour Biology and [2]Clinical Oncology, The Norwegian Radium Hospital, 0310 Oslo, Norway; [3]Institut für Experimentelle Onkologie und Transplantationsmedizin, Virchow-Klinikum, Spandauer Damm 130, D-14050 Berlin, Germany

**Summary** In a recent comparative genomic hybridization (CGH) study of a panel of sarcomas, we detected recurrent amplification of 1q21–q22 in soft tissue and bone tumours. Amplification of this region had not previously been associated with sarcoma development, but occasional amplification of *CACY/S100A6* and *MUC1* in 1q21 had been reported for melanoma and breast carcinoma respectively. Initial screening by Southern blot analysis showed amplification of *S100A6*, *FLG* and *SPRR3* in several sarcomas and, in a first attempt to characterize the 1q21–q22 amplicon in more detail, we have now investigated the amplification status of these and 11 other markers in the region in 35 sarcoma samples. *FLG* was the most frequently amplified gene, and the markers located in the same 4.5-Mb region as *FLG* showed a higher incidence of amplification than the more distal ones. However, for most of the 14 markers, amplification levels were low, and only *APOA2* and the anonymous marker D1S3620 showed high-level amplifications (> tenfold increases) in one sample each. We used fluorescence in situ hybridization (FISH) to determine the amplification patterns of two overlapping yeast artificial chromosomes (YACs) covering the region between D1S3620 and *FLG* (789f2 and 764a1), as well as two more distally located YACs in nine selected samples. Six samples had amplification of the YAC containing D1S3620 and, in three, 764a1 was also included. Five of these tumours showed normal copies of the more distal YACs; thus, it seems likely that an important gene may be located within 789f2, or very close. Two samples had high copy numbers of the most distal YACs. Taken together, FISH and molecular analyses indicate complex amplification patterns in 1q21–q22 with at least two amplicons: one located near D1S3620/789f2 and one more distal.

**Keywords:** amplification; chromosome 1; 1q21–q22; sarcomas

Cytogenetic studies have demonstrated recurrent aberrations of chromosome 1, including deletions, translocations, trisomies and amplifications, in solid tumours as well as in haematological diseases (Dracopoli et al, 1994; Weith et al, 1996). Alterations of the long arm of chromosome 1 are found both in leukaemias and in solid tumours, and are among the most common chromosomal anomalies in human neoplasia. The aberrations can be seen as trisomy of the entire long arm (Oshimura et al, 1976), as an isochromosome 1q (Kovacs, 1978) or as trisomy or duplication of a smaller region, especially 1q23–1q32 (Rowley, 1977). It has been suggested that three or more copies of a gene (or genes) in this region provide a selective advantage to cancer cells. Furthermore, the finding of partial or complete 1q trisomy being more frequent in recurrent than in primary tumours could suggest that this change may be associated with tumour progression (Weith et al, 1996).

Among solid tumours, 1q alterations had previously been reported for breast, lung and germ cell tumours. In breast cancer, comparative genomic hybridization (CGH), a method by which whole genomes may be surveyed for DNA sequence copy number changes (A Kallioniemi et al, 1992, O-P Kallioniemi et al, 1994), has demonstrated frequent gains of the whole long arm of chromosome 1 (A Kallioniemi et al, 1994; Muleris et al, 1994). However, there have been few reports on amplification of specific genes located to chromosome 1. Amplification of the *MYCL1* gene at 1p32 seems to be a common aberration in small-cell lung cancer (Nau et al, 1985; Makela et al, 1991) but has not been found in other tumour types (Dracopoli et al, 1994; Weith et al, 1996). Some melanoma cell lines show low-level amplification (or duplication) of *CACY/S100A6*[1], encoding calcyclin (Weterman et al, 1992). This gene is a member of the S100A family of calcium-binding proteins located within a cluster of S100A genes in 1q21 (Schäfer et al, 1995; Schäfer and Heizmann, 1996; Maelandsmo et al, 1997). Another gene in 1q21–q22, *MUC1*, coding for the epithelial tumour-associated antigen mucin 1 (Tsarfati et al, 1990), was found to be amplified in some breast cancers (Bieche et al, 1995; Bieche and Lidereau, 1997).

We recently studied DNA amplification in soft-tissue and bone sarcomas by CGH (Forus et al, 1995*a*,*b*) and found amplifications in about 50% of the tumours analysed. The amplicons most frequently observed were the well-characterized one at 12q13–q15 and a novel one at 1q21–q22. At the same time, gain of 1q was reported in some osteosarcomas, but was not considered a common abnormality of such tumours (Tarkkanen et al, 1995). We found that amplicons at 1q21–q22 were more frequent than those at 12q13–q15, which have been detected in a substantial number of the sarcomas analysed (Forus et al, 1993, 1994; Khatib et al, 1993; Maelandsmo et al, 1995; Nilbert et al, 1995; Berner et al, 1996). These observations could indicate that increased copy

*Received 22 May 1997*
*Revised 5 January 1998*
*Accepted 7 January 1998*

*Correspondence to:* A Forus

[1]A new logical nomenclature for this gene family has recently been approved by Genome Data Base (Schäfer et al., 1995) and will be used throughout this paper. Accordingly, *CACY* has been renamed *S100A6*.

numbers of one or more of the genes in the 1q21–q22 region could play a role in the development or progression of at least some sarcoma subtypes. Gains of 1q material have also been reported by other investigators as an occasional event in rhabdomyosarcomas (Weber-Hall et al, 1996) and more frequently in liposarcomas (Szymanska et al, 1996, 1997) and Ewing's sarcomas (Armengol et al, 1997), often involving 1q21–q22 as a minimal common region of gain. Recently, Larramendy et al (1997) reported infrequent gain of 1q material in malignant fibrous histiocytomas (MFHs), but with a more distal minimal common region, 1q24.

It might be significant that many of the tumours with amplifications of 1q are well-differentiated liposarcomas (WDLPS) and MFHs tumours that are often characterized by giant marker chromosomes containing amplified segments from various chromosomes (Heim et al, 1987; Örndal et al, 1992; Dal Cin et al, 1993; Pedeutour et al, 1993; Nilbert et al, 1994). In most WDLPS, such marker chromosomes carry segments from chromosome 12, correlating with *MDM2* amplification (Pedeutour et al, 1994), but we have recently found that some have markers that also carry chromosome 1 material, and in most of these amplification of 1q21–q22 could be demonstrated by CGH (Pedeutour et al, 1998).

In an attempt to characterize the 1q21–q22 amplicon in sarcomas, we have analysed the amplification pattern of *S100A6* and ten other markers within the same 4.5 Mb region of 1q21–q22, in addition to *MUC1* and two genes from other parts of the region, in 35 sarcomas previously studied by CGH. We have also used fluorescence in situ hybridization (FISH) to determine chromosome 1 and 2 copy numbers, as well as amplification patterns of four YAC clones from the 1q21–q22 region.

### MATERIALS AND METHODS

### Specimens

Thirty-five human sarcomas of various subtypes were analysed. In a previous study, 16 of these had been found to have amplification of 1q21–q22 by CGH, whereas the other 19 had not (Table 1)(Forus et al, 1995*a,b*). The samples were obtained directly from 21 patients with sarcomas of various subtypes, from 13 different sarcoma samples grown subcutaneously as xenografts in nude mice and from the liposarcoma cell line SW872 (ATCC). All tumours were classified according to the WHO International Histological Classification of Tumours (Schajowich, 1993; Weiss, 1994). The tissues were cut in small pieces and frozen in liquid nitrogen immediately after surgery and were stored at –70°C. Blood samples from healthy individuals were used as controls.

### Southern blot analysis

DNA extraction from tumour samples, preparation of filter blots and hybridization was performed as described previously (Forus et al, 1993). First, amplification patterns of ten different markers and genes were analysed: *FLG*, *IVL*, *SPRR3*, *SPRR1B*, *SPRR2A*, *S100A6* (*CACY*) and *S100A2* (*S100L*), all of which are physically mapped within 2 Mb and are part of a 6-Mb YAC contig in 1q21 (Marenholz et al, 1996), as well as two markers that have been mapped distal to the 6-Mb contig in the order *CRP–APOA2* and *MUC1* (Weterman et al, 1996; Chromosome 1 www page http://linkage.rockefeller.edu/chr1/).

Based on the results obtained with these probes, we later included some additional anonymous clones that map proximal to *FLG* (D1S3620 and D1S3623) and distal to *S100A2* (D1S3625 and D1S3628) (Marenholz et al, 1996).

Southern blots were sequentially hybridized to probes from each locus and to a control probe from chromosome 2 (*APOB*) (Huang et al, 1985). Quantitation of signal intensity was achieved by two-dimensional densitometry on a Molecular Dynamics laser densitometer. The net signals from specific bands were corrected for unequal sample loading by calibration relative to the signal obtained with the *APOB* control probe. Mean signals from three (or more) different blots were used to measure the amplification levels in the tumour. As the percentage of tumour cells versus normal cells for each sample was not known, the actual amplification levels in the tumour cells may be higher than the presented values.

The signals were compared with signals from control samples with a normal karyotype (leukocytes) and interpreted as described. Borderline amplification – a signal two- to threefold more intense than signals from normal samples (i.e. average probe/APOB ratio for tumour divided by average probe/APOB ratio for normal sample between 2.0 and 2.9); low level – three- to fivefold increase (tumour–normal value between 3.0 and 4.9); moderate – five- to tenfold increase (tumor–normal value between 5.0 and 9.9); and high – > tenfold increase (tumour–normal value 10 or higher).

### Fluorescent in situ hybridization (FISH) to interphase nuclei

#### *Preparation of interphase nuclei*

Frozen tumour tissue was pulverized in liquid nitrogen, transferred to a centrifuge tube and immediately fixed in 3:1 methanol–acetic acid. After centrifugation, the pellet was resuspended in 60% acetic acid. Two or three drops of the suspension were applied onto slides (Menzel Superfrost) prewarmed to 45–50°C, and left to dry at the same temperature. Slides were stored at –20°C before use.

#### *Preparation of probes*

YAC DNA was labelled with biotin-14-dATP or digoxygenin-11-dUTP (Boehringer Mannheim, Germany) by nick translation (GibcoBRL Life Technologies, USA). For each hybridization, 200–300 ng of labelled YAC DNA was prehybridized with a 50- to 100-fold excess of human Cot-1 DNA and 2–5 µg of yeast DNA, whereas biotin- or digoxygenin-labelled centromere probes were simply premixed with human placenta DNA (Sigma, St. Louis, MO, USA). Subsequently, the probes were dissolved in 50% formamide/10% dextran sulphate/0.3 M sodium chloride, 30 mM sodium citrate (2 × SSC).

#### *In situ hybridization*

Slides were thawed and immersed in 75% ethanol at 4°C for 1–2 h before use, air dried and denatured in 70% formamide/2 × SSC, pH 8.0 for 3 min at 74°C, washed three times in ice cold 2 × SSC, dehydrated in ethanol (70%, 90%, 96% and 100%) and air dried. Thereafter, slides were treated with proteinase K (0.1 µg $ml^{-1}$ in 20 mM Tris-HCl/2 mM calcium chloride, pH 7.0) for 10 min at room temperature, washed in 2 × SSC, dehydrated and air dried. Probes were denatured for 10 min at 80°C, prehybridized for 15–30 min at 37°C and applied to slides at room temperature. Hybridization was done overnight at 37°C. After hybridization, the slides were washed three times for 10 min in 50% formamide/ 2 × SSC at 45°C and then three times for 10 min in 2 × SSC at

© Cancer Research Campaign 1998

Table 1 Histopathological characteristics and 1q21–q22 amplification data of the 35 sarcomas analysed

| Tumour | Histological subtype | Sample from | Histological grade | Location | Preoperative treatment | Amplification by CGH | Amplification Mol probes |
|---|---|---|---|---|---|---|---|
| LMS2x | | Rec | 3 | Arm | No | 1q21–q23 | Yes |
| LMS14 | | Prim | 4 | Lung | No | 1q21–q23 | Yes |
| LMS15 | | Prim | 3 | Abdomen | No | 1q21–q23 | Yes |
| LS2 | Well-differentiated | Prim | 1 | Thigh | No | 1q21–q22 | Yes |
| LS3x | Pleomorphic | Prim | 4 | Abdomen | No | 1q21–q22 | Yes |
| LS5x | Round cell | Prim | 4 | Abdomen | No | No | – |
| LS6 | Well-differentiated | Prim | 1 | Gluteal | No | 1q21–q22 | Yes |
| LS7 | Sclerosing/well-differentiated | Prim | 2 | Paraspinal | No | No | – |
| LS9 | Well-differentiated/lipoma like | Prim | 1 | Thigh | No | 1q21–q22 | Yes |
| LS10 | Undifferentiated | Cell line | | | | No | – |
| LS13 | Well-differentiated | Prim | 1 | Thigh | No | 1q21–q22 | Yes |
| LS15 | Myxoid/round cell | Prim | 4 | Gluteal | No | No | – |
| LS18 | Round cell | Prim | 4 | Abdomen | No | No | – |
| LS21 | Well-differentiated | Prim | 2 | Abdomen | No | 1q21–q22 | Yes |
| LS22 | Various differentiation | Rec | 3 | Abdomen | Chemotherapy | No | Yes |
| LS26 | Myxoid/round cell | Prim | 3 | Groin | No | No | – |
| LS28 | Pleomorphic/round cell | Prim | 4 | Abdomen | No | No | – |
| LS32 | Mixed | Prim | 3 | Thigh | No | No | – |
| MFH3x | | Prim | 4 | Retroperitoneal | No | No | Yes |
| MFH19 | | Rec | 4 | Thigh | No | 1q21–q22/23 | Yes |
| MFH21 | | Prim | 4 | Thorax | No | 1q21–q22 | Yes |
| MFH25 | | Prim | 4 | Thigh | No | No | – |
| MFH36 | | Prim | 4 | Shoulder | No | 1pter–q22 | Yes |
| MS2x | | Prim | 3/4 | Leg | No | No | – |
| MS8x | | Prim | 3 | Gluteal | No | 1q21–q22 | Yes |
| OS4x | | Prim | 4 | Femur | Chemotherapy | 1q11–q23 | Yes |
| OS6x | | Prim | 4 | Femur | Chemotherapy | No | – |
| OS7x | | Prim | 4 | Femur | Chemotherapy | No | – |
| OS8x | | Met | 4 | Femur | Chemotherapy | No | – |
| OS9x | | Prim | 3/4 | Femur | No | 1q21–q25 | Yes |
| OS11x | | Prim | 4 | Femur | No | No | – |
| OS13x | | Met | 4 | Lung | Chemotherapy[a] | 1q21–q23 | Yes |
| OS21 | | Prim | 4 | Thigh | Chemotherapy | No | Yes |
| OS29 | | Prim | 4 | Pelvis | Chemotherapy[b] | No | – |
| PNET1 | | Prim | 4 | Leg | No | No | – |

LMS, leiomyosarcoma; LS, liposarcoma; MFH, malignant fibrous histiocytoma; MS, malignant peripheral nerve sheath tumour (malignant schwannoma); OS, osteosarcoma; PNET = primitive neuroectodermal tumour. For the liposarcomas, the histological subtype is indicated if known. The stage, histological grade and localization of the tumour sample analysed are indicated. Prim, primary tumour; Rec, recurrent; Met, metastasis. For each sample it is indicated whether amplification of 1q21–q22 could be demonstrated by CGH and/or molecular analysis (Mol probes, > twofold increase in gene dosage). LS10 is a cell line. Some of the patients received chemo- or radiotherapy before surgery. [a]This patient received adjuvant chemotherapy after the primary tumour, which was excised 4 years before the removal of the sample analysed here. [b]This was a therapy-induced osteosarcoma; the patient had Ewing's sarcoma 5 years before this tumour and received chemo- and radiotherapy.

60°C. For detection, we used fluorescein isothiocyanate (FITC)-conjugated anti-digoxygenin (Boehringer Mannheim), avidin-conjugated Texas Red (Vector Laboratories, Burlingame, CA, USA) or avidin-conjugated CY3 (Amersham Life Science, Little Chalfont, UK). The interphase nuclei were counterstained with 4′,6-diamino-2-phenylindole (DAPI) and mounted in anti-fade solution (Vector Laboratories).

### Evaluation of results

Hybridized slides were examined visually using a Zeiss Axioplan microscope equipped with appropriate single-bypass filters for exitation of DAPI, FITC, Texas Red and rhodamine/CY3, and double bypass filters for excitation of DAPI/rhodamine and DAPI/FITC. The slides were manually scanned at 63× or 100× magnification with DAPI excitation to localize the interphases. Nuclei that were either partially or totally overlapping or not intact were not analysed. For each probe, the number of spots was counted in at least 150 nuclei.

### Probes

### cDNA and genomic probes

The following probes were used: pHX5 *FLG* (Presland et al, 1992), containing a part of the coding region from the 3′ end of the human filaggrin gene, kindly provided by Drs Fleckman and Presland; *IVL* (pI-2) (Eckert and Green, 1986) containing the 3′ end cDNA of human involucrin, kindly provided by Drs Easley and Green; cDNA probes for the genes *SPRR1B*, *SPRR2A* and *SPRR3* (Gibbs et al, 1993; Hohl et al, 1995), kindly provided by Dr Backendorf; a cDNA clone for human calcyclin (*CACY/S100A6*)(pMW1)(Weterman et al, 1992), kindly provided by Dr Bloemers, and CAN19; a near full-length cDNA probe for the human *S100L/S100A2* (Lee et al, 1992), kindly provided by Dr Sager. All these probes are located within the same 2-Mb region in 1q21 (Mahrenholz et al, 1996). Three other genes from 1q21–q22 were also checked: pCRP-5, a cDNA clone for human C-reactive protein (CRP) (Tucci et al, 1983); cDNA for apolipoprotein AII (*APOA2*) (Rogne et al, 1989); and pMUC10, a genomic clone for

© Cancer Research Campaign 1998

**Figure 1** Representative Southern blot hybridizations demonstrating the amplification pattern of the *FLG*, *SPRR1B*, *SPRR2A*, *SPRR3* and *S100A2* loci (**A**), D1S3620 and D1S3623 (**B**) and *APOA2* (**C**). DNA from each sample was digested with *Hin*dIII and sequentially hybridized to probes as indicated to the right. Leucocyte DNA was included as a control for normal copy number, and a probe for *APOB* was used to calibrate for unequal sample loading. An 'A' below a panel indicates that the corresponding gene is amplified more than threefold, an (**A**) indicates borderline amplification (signal two to three times more intense than the signal from normal DNA). LS, liposarcoma; OS, osteosarcoma; MFH, malignant fibrous histiocytoma; MS, malignant peripheral nerve sheath tumour (malignant schwannoma)

the epithelial tumour-associated antigen *MUC1* (Swallow et al, 1987) (UK DNA probe bank). Anonymous probes from the 1q21 region that are located approximately 4 Mb apart were: D1S3620 and D1S3623, which are centromeric to *FLG*, and D1S3625 and D1S3628, which are telomeric to *CACY/S100A6* (Marenholz et al, 1996). A cDNA probe for the *APOB* gene on human chromosome 2, kindly provided by Dr Breslow (Huang et al, 1985), was used to calibrate for unequal sample loading.

### Centromere probes

The centromere probes used were biotin or digoxygenin-labelled human chromosome 1 α-satellite (D1Z5) and human chromosome 2 α-satellite (D2Z) (Oncor, Gaithersburg, MD, USA).

### Yeast artificial chromosome (YAC) clones

We used two YACs from the Centre D'Etude du Polymorphisme Humain (CEPH) mega-YAC library, 789f2 and 764a1 (Marenholz et al, 1996). These YACs are part of a 6-Mb contig in 1q21 and cover the region from *FLG* to D1S3620 (Marenholz et al, 1996). The two other YACs, 935b12 and 883h6, were from the CEPH library described by Albertsen et al (1990) and have been mapped to the 1q21–q22/q23 region (CEPH-Genethon map).

### RESULTS

### Southern blot analysis

Initial screening of selected tumour samples using probes for *S100A6*, *FLG* and *SPRR3*, which are located in the same 2-Mb region of 1q21–q22, detected amplifications at various levels in all the tumours tested and, therefore, these and four other markers in the region were analysed in more samples. We also included *MUC1*, as this gene has been reported to be amplified in breast cancer, and two other genes in the region, *CRP* and *APOA2*, for which probes were available.

All the probes from 1q21–q22 detected amplification in some of the samples (Figures 1 and 2). Nine samples had more than a threefold increase in gene dosage of one or more of the genes

 © Cancer Research Campaign 1998

**Figure 2** Amplification of 1q21–q22 in human sarcomas. The upper part of the figure shows a composite map of 1q21–q22 adapted from physical (Marenholz et al, 1996; Schäfer and Heizmann, 1996) and genetic (Dracopoli et al, 1994; Weterman et al, 1996) mapping data. Loci are listed in their order from the centromeric (left) to the telomeric (right) side. An empty column indicates > 200 kb distance between two loci. Between *S100A2* and *S100A6* there are at least three additional S100A genes that have not been tested in this study (Schäfer et al, 1995; Schäfer and Heizmann, 1996). The physical distances between *S100A6*, *CRP* and *APOA2* are not known, but the genetic distance between *S100A6* and *CRP* is around 9 cM (Murray et al, 1994), as discussed by Mahrenholz et al (1996). *MUC1* has not been located relative to the other genes, but linkage analysis gives the order (centromere to telomere) *MUC1–CRP–APOA2* (Dracopoli et al, 1994; Weith et al, 1996). The densitometrically determined levels of amplification are divided into four categories as indicated, based on average signals from at least three different blots. Tumour types and numbers are given to the left [LMS, leiomyosarcoma; LS, liposarcoma; MFH, malignant fibrous histiocytoma; MS, malignant peripheral nerve sheath tumour (malignant schwannoma); OS, osteosarcoma]. The number of samples with amplification (two to three fold increase or more) of each locus is listed below. The CGH column lists the previously detected 1q21–q22 amplifications (Forus et al, 1995*a,b*). A '–' sign = no 1q21–q22 amplification detected by CGH. ND, not done

(Figure 2) and ten other samples showed borderline amplification (two- to threefold increase). Among these were three samples in which no gain of 1q21–q22 was detected by CGH (LS22, MFH3× and OS21). The remaining 16 tumours had a normal copy number of all the genes tested.

*FLG*, encoding human epidermal profilaggrin (Presland et al, 1992), was the most frequently amplified gene, but in most samples only borderline amplification of this gene was found. The genes localized close to FLG (*IVL*, *SPRR3*, *SPRR1B*, *SPRR2A*, *S100A6* and *S100A2*), were amplified in fewer samples and were also at borderline or low levels in most cases. However, *SPRR3*, *SPRR2A*, *S100A6* and *S100A2* showed amplification levels above fivefold in some samples (LMS2x, LS13 and MS8x). Three samples had amplification of all seven genes at variable levels (Figure 2, LMS2x, LS2 and LS13). Among the loci that has not been mapped to the above-mentioned 6-Mb contig, *CRP* was the only one that was included in the amplicon in LS6, and *APOA2* was the only gene amplified above tenfold in one of the liposarcomas (LS21). *MUC1* was amplified in five samples, i.e. in fewer cases than any of the other genes tested.

The region between *FLG* and *S100A2* was more frequently amplified than loci located telomeric to this interval (*MUC1*, *APOA2* and *CRP*). We therefore determined the amplification patterns also of some additional anonymous clones that map proximal to *FLG* (D1S3620 and D1S3623) and distal to *S100A2* (D1S3625 and D1S3628), delineating a 4.5-Mb region in 1q21–q22 (Marenholz et al, 1996). Copy numbers of these probes were determined in those 19 samples that had previously revealed amplification of one or more of the genes. As shown in Figure 2, D1S3623, located proximal but close to *FLG* (Marenholz et al, 1996), was as frequently amplified as *FLG*, whereas the more proximal marker, D1S3620, was amplified in fewer cases. D1S3620 was highly amplified in MS8x, whereas D1S3623 and *FLG* showed normal copy numbers, but the sample had another amplified cluster covering *IVL* through *SPRR2A*. D1S3625 and D1S3628, localized distal to *S100A2*, showed a similar amplification pattern to *S100A2*.

### FISH analysis of chromosome 1 and 2 copy numbers

Although the variable gene dosages observed along the chromosome were consistent with regional low-level amplification, we wanted to ascertain that the tumours did not have extra copies of chromosome 1 or abnormal ratios between chromosome 1 and

© Cancer Research Campaign 1998 

Table 2 Selected tumour samples analysed by FISH using centromere probes and YACs in 1q21–q22

| Tumour | Centromere analysis | | | YAC analysis | | | |
|---|---|---|---|---|---|---|---|
| | No. 1 | No. 2 | (No. 1: No. 2) | 789f2 | 764a1 | 935b12 | 883h6 |
| LS2 | 2.3 | 2.1 | 1.1 | **43%** | **43%** | 7% | 5% |
| LS3x | 2.0 | 2.0 | 1.0 | <u>33% (3–4s)</u> | <u>43% (3–4s)</u> | **83%** | **73%** |
| LS6 | 2.1 | 2.0 | 1.1 | **47%** | *11% (3–4s)* | 3% | 5% |
| LS13 | 2.2 | 2.2 | 1.0 | **51%**<br>4.5% (4–6s) | **55%** | 4% | 5% |
| LS21a | 2.2 | 2.1 | 1.0 | **46%**<br>*18% (4–6s)* | *16% (3–4s)* | <u>38% (3–4s)</u> | **46%**<br>*18% (4–6s)* |
| LS21b | 2.2 | 2.1 | 1.0 | 0% | 2% (4s) | 0% | 0% |
| LS22 | 4.2 | 4.0 | 1.1 | NA | NA | NA | NA |
| MFH3x | 5.7 | 5.8 | 1.0 | NA | NA | NA | NA |
| MFH25 | 2.2 | 2.1 | 1.1 | 5.7% | 5% | 1% | 1% |
| MFH36 | 2.4 | 2.1 | 1.1 | **75%** | **75%** | *19.5% (3–4s)* | *16% (3–4s)* |
| MS2x | 2.2 | 2.2 | 1.0 | 5% | 2% | 1.5% | 0.5% |
| MS8x | 2.0 | 2.0 | 1.0 | **85%** | 6% | *18% (3–4s)* | 3% |
| OS4x | 4.5 | 5.1 | 0.9 | NA | NA | NA | NA |

Centromere 1 and 2 as well as four YAC clones from 1q21–q22/q23 have been analysed by FISH in selected tumours with amplifications in 1q21–q22 detected by CGH and/or molecular analysis and in two samples with normal copy numbers of the genes (MS2x and MFH25). For each sample, at least 150–200 nuclei were counted. YACs 789f2 and 764a1 are partly overlapping. YACs 935b12 and 883h6 are located very close together, but more distal (not shown). **Centromere analyses:** for each sample, the average centromere counts are shown. All tumours had a balanced chromosome 1 : 2 ratio, but LS22, MFH3x and OS4x had abnormal (increased) chromosome numbers and were not analysed with the YACs (NA). **YAC analyses:** for each sample, the percentage of nuclei with abnormal copy numbers is shown. Bold text: high copy number gains of a YAC (at least ten signals). Underlined text: three to four YAC signals in more than 30% of the nuclei. In two samples with amplification of 789f2 (LS13) and 789f2 and 883h6 (LS21), a fraction of the nuclei showed four to six signals, as indicated by split boxes. Italic: 10–20% of the nuclei showed three to four signals. In addition, some samples gave three to four signals with one or two of the YACs tested in less than 10% of the nuclei. Notably, in LS21, two different parts of the tumour (a, b) showed different amplification patterns.

chromosome 2 (used for normalization). We therefore determined the copy numbers of centromeres 1 and 2 by interphase FISH on nuclei from ten of the samples with amplicons detected by molecular analysis and/or CGH, as well as two tumours with apparently normal copy numbers of all the markers tested (MS2x and MFH25[2]). All the samples tested had similar mean numbers of centromere 1 and 2 per nucleus (Table 2). Seven of the tumours showed normal centromere counts in most of the nuclei, whereas three (LS22, MFH3x and OS4x) had four to six copies of each.

### FISH analysis of YAC copy numbers

The molecular results gave no clear indications as to whether the region studied was likely to contain the core of the amplicon, although the high amplification levels of D1S3620 found in MS8x could indicate that this marker was close. The region between D1S3620 and *FLG* has not been mapped in detail, but two YACs that cover this region, 789f2 and 764a1, were available [the positions of these YACs was mapped by Mahrenholz et al (1996) and are indicated in Figure 2]. We studied the amplification pattern of these YACs only in samples with normal average cen 1 and cen 2 copy numbers, including seven tumours with amplifications in this region and two control samples (MS2x and MFH25). The samples with increased centromere 1 and 2 copies were excluded from the study because it would be very difficult to obtain reliable results. Two additional YACs were selected from the CEPH-Genethon map of region 1q21–q23 and mapped by FISH analysis distal to 764a1 (not shown). However, FISH on normal metaphases could not determine whether 935b12 or 883h6 is the most distal YAC as they gave overlapping signals. Results from FISH analyses with these four YACs are shown in Table 2 and Figure 3.

As shown in Table 2, 789f2, covering D1S3620, detected high copy numbers (more than ten, but in most samples uncountable) in nuclei from five of the samples and three to four signals in one (LS3x). However, there was considerable heterogeneity among the nuclei. In MFH36 and MS8x, a major fraction of the nuclei (75% or more) exhibited amplification; in the other samples, LS2, LS3x, LS6 and LS13, only about 50% or less. 764a1, covering D1S3623, detected high copy numbers in three samples and three or four signals in one, but, except for MFH36, only about 50% of the nuclei or less had these aberrations. The more distal YACs were amplified in fewer samples. 883h6 and 935b12 detected high copy numbers only in LS3x, involving more than 70% of the nuclei.

We analysed two different pieces from the last sample, LS21. As shown in Table 2, considerable heterogeneity was detected: one part of the tumour had no amplifications, whereas in the other part 789f2 and 883h6 showed high copy numbers. In addition, a fraction of the nuclei also showed four to six signals with these two YACs. 789f2 and 883h6 were always co-amplified in this part of the tumour, but only in 46% of the nuclei analysed. The other YACs, 764a1 and 935b12, detected three or four signals in 16% and 38% of the nuclei respectively.

For some samples, one or two of the YACs tested gave three or four signals in a smaller fraction of the cells (less than 20%). As all these samples showed higher amplification levels with at least one of the other YACs, these aberrations were considered to be of less importance.

### DISCUSSION

Gain of chromosomal sequences, or amplification, is frequently found in human cancers. Functional analysis and clinical observations have supported the hypothesis that amplification and

[2]MFH25 was called MFH43 in a previous study (Forus et al, 1995*a*).

 © Cancer Research Campaign 1998

**Figure 3** FISH analysis using YACs from 1q21–q22/q23 on interphase nuclei from MFH36, MS8x and LS2. (**A–C**) interphase nuclei from MFH hybridized with (**A**) digoxygenin (dig)-labelled 789f2 detected by FITC (in green) and biotin-labelled 764a1 detected by avidin-CY3 (in red), (**B**) biotin-labelled 883h6 and (**C**) 935b12, detected by avidin-Cy3 (in red). YAC 789f2 and 764a1 detect amplification and were always co-amplified, but 789f2 is amplified at higher levels (**A**). 883h6 and 935b12 show normal copy numbers in most of the nuclei (883h6 gives three signals in one). (**D** and **E**) interphase nuclei from MS8x hybridized with biotin-labelled 789f2 (**D**) and 935b12 (**E**). 789f2 detects high-level amplification whereas 935b12 gives normal signals. (**F**) interphase nucleus from LS2 hybridized with digoxygenin (dig)-labelled 789f2 (in green) and biotin-labelled 764a1 (in red). The YACs were always co-amplified, but also, here, 789f2 is amplified at higher levels overexpression of cellular oncogenes are important for tumorigenesis or tumour progression (Alitalo and Schwab, 1986). In its simplest form, one might expect that amplification and overexpression of a single dominant (proto)oncogene could provide a selective growth advantage to the tumour cells. If so, all amplicons would contain this 'driver' gene and its closely flanking markers and other amplified sequences would only be random passengers of the amplicon. However, studies of 11q13 amplification in breast cancer and 12q13–15 amplification in sarcomas have revealed more complex situations with multiple amplification units and several candidate genes in the same region (Gaudray et al, 1992; Berner et al, 1996; Wolf et al, 1997).

We have previously demonstrated frequent amplification of the 12q13–15 segment, including the *CDK4*, *MDM2* and *HMGIC* genes, in human sarcomas (Forus et al, 1993; Maelandsmo et al, 1995; Berner et al, 1996, 1997). In addition, CGH analysis detected amplification of the 1q21–q22 region in even more samples from this tumour panel (Forus et al, 1995*a*, *b*), indicating that 1q21–q22-located genes may also play an important role in the development and/or progression of such tumours. Similar observations have also been reported by other investigators (Szymanska et al, 1996; Armengol et al, 1997).

In this first molecular analysis of the 1q21–q22 amplifications in sarcomas, we determined the amplification status of 11 markers located in a region covering 4.5 Mb, including *S100A6*, which had been reported to be amplified in melanomas (Weterman et al, 1992). We also analysed three more distal markers, including *MUC1*, amplified in some breast carcinomas (Bieche and Lidereau, 1997; Bieche et al, 1995).

One would expect that markers closest to the 'core' of the amplicon would be amplified most frequently and at the highest levels. The most frequently amplified markers were moderately increased in most of the samples, and only *APOA2* and the anonymous marker D1S3620 were amplified more than tenfold in one sample each (in LS21 and MS8x respectively). Thus, these criteria were not fulfilled for any of the markers tested here. The relevance of the moderate copy number increases is unclear at present, but it seems likely that they are due to amplification processes within the segment, probably selected for by one or more unknown oncogene(s) in the region.

As D1S3620 detected high-level amplification in one sample, we analysed the region around this marker in more detail by FISH in nine selected samples, using two partly overlapping YACs covering sequences between D1S3620 and *FLG*. YAC 789f2, which includes D1S3620 and extends towards D1S3623 (Figure 2), detected high copy numbers in six of these nine tumours (LS2, LS6, LS13, LS21, MFH36 and MS8x, Table 2), but in only one part of LS21. The more distal 764a1 was amplified in only three of these tumours (LS2, LS13, and MFH36). No YACs near *APOA2*, the other region with high-level amplification, were available, but we analysed the amplification pattern of 935b12 and 883h6, which we have mapped distal to 764a1 (Table 2). Like *APOA2*, both YACs detected amplification in LS3x. In LS21, showing the highest copy numbers of *APOA2*, only one part of the tumour showed amplification of 883h6, again indicating tumour heterogeneity.

In five of the samples with amplification of 789f2, the more distal YACs (935b12 and 883h6) showed normal copy numbers in most of the nuclei. These results could suggest that in a subset of

© Cancer Research Campaign 1998

tumours a target gene is located within 789f2, or even more proximal, whereas more distal sequences seem to be of less importance. In LS3x, on the other hand, the focus of the amplicon seems to be more distal, i.e. in the region of 883h6, 935b12 and *APOA2*, whereas LS21 may have at least two amplicons, one near 789f2 and another one in the region of *APOA2* and 883h6. The presence of multiple amplified regions on the 1q arm in sarcomas has been shown previously (Forus et al, 1995*a*).

It is somewhat puzzling that for most samples the high-level amplifications detected by FISH were often found only in about 50% of the nuclei. One possibility is that the cells with normal copy numbers in tumours with amplification are normal cells, in which case the amplification levels determined from the Southern analyses would be underestimated. However, such a large fraction of normal cells would be surprising, e.g. in the homogeneous WDLPS samples. Conversely, the variation in copy numbers between the nuclei could be an indication of clonal variations within the tumours and, then, Southern analysis would only reveal the average copy numbers. This interpretation is supported by the heterogeneous amplification status detected in some of the tumours (e.g. LS21). It seems likely that the results shown here (Table 2) reflect tumour heterogeneity as well as, to some extent, the presence of normal cells. Tumour cell percentage in the different pieces was not evaluated before DNA isolation, therefore we do not know to what extent the presence of normal cells may have affected the measured amplification levels.

Most of the samples analysed were from primary tumours of histological grade 3 or 4 (Table 1), and there is no correlation between malignancy grade or any other known clinical parameters and the presence of 1q21–q22 amplifications. Many of the patients with osteosarcoma have received chemotherapy before surgery, but the presence of the 1q21–q22 amplicon and preoperative treatment is not correlated. Therefore, it seems unlikely that such amplifications could be therapy induced. As the 1q21–q22 amplicon is present in primary as well as in recurrent and metastatic samples, it is possible that amplification of oncogenes in this region plays a role in the development of these tumours but may be less important for progression and metastasis.

The previous CGH analyses of this tumour panel (Forus et al, 1995*a*,*b*), as well as other studies of osteosarcomas and liposarcomas (Tarkkanen et al, 1995; Szymanska et al, 1996), indicate that 1q21–q22 amplifications are more frequent than those at 12q13–q15 and thus may be of great importance. In this first molecular characterization of the 1q21–q22 amplifications in sarcomas, we observed frequent but relatively low copy number increases for most of the markers tested, but also some high-level amplifications. Taken together, our results indicate more than one core of the amplicon and further analyses are required to determine important sequences more precisely and to find the relevant genes.

### ACKNOWLEDGEMENTS

This work was supported by the Norwegian Cancer Society and by the European Commission through the Biomedicine and Health project 'Integrated analysis of expression and chromosomal organization of genes localized on human chromosome 1q21: implications for human disease and cancer' under BIOMED 2 (BMH4-CT96-0319). We are grateful to Drs Helgerud and Høie for providing the clinical samples, Dr Stenwig for histological classification of the tumours, Ingo Marenholz (Berlin) and Dr Bjerkehagen (Oslo) for valuable advice and support and Kjetil Boye Pedersen for excellent technical assistance.

### REFERENCES

Albertsen HM, Abderrahim H, Cann HM, Dausset J, Le Paslier D and Cohen D (1990) Construction and characterisation of a yeast artificial chromosome library containing seven haploid human genome equivalents. *Proc Natl Acad Sci USA* **87**: 4256–4260

Alitalo K and Schwab M (1986) Oncogene amplification in tumor cells. *Adv Cancer Res* **47**: 235–281

Armengol G, Tarkkanen M, Virolainen M, Forus A, Valle J, Böhling T, Asko-Seljavaara S, Blomquist C, Elomaa I, Karaharju E, Kivioja AH, Siimes MA, Tukiainen E, Caballin MR, Myklebost O and Knuutila S (1997) Recurrent gains of 1q8 and 12 in the Ewing family of tumours by comparative genomic hybridisation. *Br J Cancer* **75**: 1403–1409

Berner J-M, Forus A, El Kahloun A, Meltzer PS, Fodstad Ø and Myklebost O (1996) Separate amplified regions encompassing CDK4 and MDM2 in human sarcomas. *Genes Chrom Cancer* **17**: 254–259

Berner J-M, Meza-Zepeda LA, Kools PFJ, Forus A, Schoenmakers EFPM, Van de Ven WJM, Fodstad Ø and Myklebost O (1997) HMGIC, the gene for an architectural transcription factor, is amplified and rearranged in a subset of human sarcomas. *Oncogene* **14**: 2935–2941

Bieche I and Lidereau R (1997). A gene dosage effect is responsible for high overexpression of the MUC1 gene observed in human breast tumours. *Cancer Genet Cytogenet* **98**: 75–80

Bieche I, Champeme M-H and Lidereau R (1995) Loss and gain of distinct regions of chromosome 1q in primary breast cancer. *Clin Cancer Res* **1**: 123–127

Dal Cin P, Kools P, Sciot R, De Wever I, Van Damme B, Van de Ven W and Van den Berghe H (1993) Cytogenetic and fluorescence in situ hybridization investigation of ring chromosomes characterizing a specific pathologic subgroup of adipose tissue tumors. *Cancer Genet Cytogenet* **68**: 85–90

Dracopoli NC, Bruns GAP, Brodeur GM, Landes GM, Matise TC, Seldin MF, Vance JM and Weith A (1994) Report of the first international workshop on human chromosome 1 mapping 1994. *Cytogenet Cell Genet* **67**: 144–165

Eckert RL and Green H (1986) Structure and evolution of the human involucrin gene. *Cell* **46**: 583–589

Forus A, Flørenes VA, Maelandsmo GM, Meltzer PS, Fodstad Ø and Myklebost O (1993) Mapping of amplification units in the q13–14 region of chromosome 12 in human sarcomas: some amplica do not include MDM2. *Cell Growth Differ* **4**: 1065–1070

Forus A, Flørenes VA, Maelandsmo GM, Fodstad Ø and Myklebost O (1994) 12q13–14 amplica in human sarcomas without MDM2 include CDK4, SAS and GADD153/CHOP. *Cancer Genet Cytogenet* **77**: 200

Forus A, Olde Weghuis D, Smeets D, Fodstad Ø, Myklebost O and Geurts van Kessel A (1995*a*) Comparative genomic hybridization analysis of human sarcomas. I. Occurrence of genomic imbalances and identification of a novel major amplicon at 1q21–q22 in soft tissue sarcomas. *Genes Chrom Cancer* **14**: 8–14

Forus A, Olde Weghuis D, Smeets D, Fodstad Ø, Myklebost O and Geurts van Kessel A (1995*b*) Comparative genomic hybridization analysis of human sarcomas. II. Identification of novel amplicons at 6p and 17p in osteosarcomas. *Genes Chrom Cancer* **14**: 15–21

Gaudray P, Szepetowski P, Escot C, Birnbaum D and Thelliet C (1992) DNA amplification at 11q13 in human cancer: from complexity to perplexity. *Mutat Res* **276**: 317–328

Gibbs S, Fijneman R, Wiegant J, Geurts van Kessel A, van de Putte P and Backendorf C (1993) Molecular characterization and evolution of the SPRR family of keratinocyte differentiation markers encoding small proline-rich proteins. *Genomics* **16**: 630–637

Heim S, Mandahl N, Kirstoffersson U, Mitelman F, Rooster B, Rydholm A and Willen H (1987) Marker ring chromosome: a new cytogenetic abnormality characterizing lipogenetic tumors. *Cytogenet Cell Genet* **24**: 319–326

Hohl D, de Viragh PA, Amiguet-Barras F, Gibbs S, Backendorf C and Huber M (1995) The small proline-rich proteins constitute a multigene family of differentially regulated cornified cell envelope processor proteins. *J Invest Dermatol* **104**: 902–909

Huang LS, Bock SC, Feinstein SI and Breslow JL (1985) Human apolipoprotein B cDNA clone isolation and demonstration that liver apolipoprotein B mRNA is 22 kilo bases in length. *Proc Natl Acad Sci USA* **82**: 6825–6829

Kallioniemi A, Kallioniemi O-P, Sudar D, Rutovitz D, Gray JW, Waldman F and Pinkel D (1992) Comparative genomic hybridization for molecular cytogenetic analysis of solid tumors. *Science* **258**: 818–821

Kallioniemi A, Kallioniemi O-P, Piper J, Tanner M, Stokke T, Chen L, Smith HS, Pinkel D, Gray JW and Waldman FM (1994) Detection and mapping of amplified DNA sequences in breast cancer by comparative genomic hybridization. *Proc Natl Acad Sci USA* **91**: 2156–2160

© Cancer Research Campaign 1998

Kallioniemi O-P, Kallioniemi A, Piper J, Isola J, Waldman FM, Gray JW and Pinkel D (1994) Optimizing comparative genomic hybridization for analysis of DNA sequence copy number changes in solid tumors. *Genes Chromosom Cancer* **10**: 231–243

Khatib ZA, Matsushime H, Valentine M, Shapiro DN, Sherr CJ and Look AT (1993) Coamplification of the CDK4 gene with MDM2 and GLI in human sarcomas. *Cancer Res* **53**: 5535–5541

Kovacs G (1978) Abnormalities of chromosome no.1 in human solid malignant tumours. *Int J Cancer* **1**: 688–694

Larramendy ML, Tarkkanen M, Blomqvist C, Virolainen M, Wiklund T, Asko-Seljavaara S, Elomaa I and Knuutila S (1997) Comparative genomic hybridization of malignant fibrous histiocytoma reveals a novel prognostic marker. *Am J Pathol* **151**: 1153–1161

Lee SW, Tomasetto C, Swisshelm K, Keyomarsi K and Sager R (1992) Down-regulation of a member of the S100 gene family in mammary carcinoma cells and re-expression by azadeoxycytidine treatment. *Proc Natl Acad Sci USA* **89**: 2504–2508

Maelandsmo GM, Berner J-M, Flørenes VA, Forus A, Hovig E, Fodstad Ø and Myklebost O (1995) Homozygous deletion frequency and expression levels of the CDKN2 gene in human sarcomas – relationship to amplification and mRNA levels of CDK4 and CCND1. *Br J Cancer* **72**: 393–398

Maelandsmo GM, Flørenes VA, Mellingsaeter T, Hovig E, Kerberl RS and Fodstad Ø (1997) Differential expression patterns of S100A2, S100A4 and S100A6 during progression of human malignant melanoma. *Int J Cancer* **74**: 464–469

Makela TP, Kere J, Winquist R and Alitalo K (1991) Intrachromosomal rearrangements fusing L-myc and rlf in small-cell lung cancer. *Mol Cell Biol* **11**: 4015–4021

Marenholz I, Volz A, Ziegler A, Davies A, Ragoussis I, Korge BP and Mischke D (1996) Genetic analysis of the epidermal differentiation complex (EDC) on human chromosome 1q21: chromosomal orientation, new markers and a 6 Mbp YAC contig. *Genomics* **37**: 295–302

Muleris M, Almeida A, Gerbault-Seureau M, Malfoy B and Dutrillaux B (1994) Detection of DNA amplification in 17 primary breast carcinomas with homogeneously staining regions by modified comparative genomic hybridization technique. *Genes Chromosom Cancer* **10**: 160–170

Murray JC, Buetow KH, Weber JL, Ludwigsen S, Scherpbier-Heddema T, Manion F, Quillen J, Sheffield VC, Sunden S, Dukyk GM (Cooperative Human Linkage Center); Weissenbach J, Gyapay G, Dib C, Morrissette J, Lathrop GM, Vignal (Généthon); White R, Matsunami N, Gerken S, Melis R, Alberten H, Plaetke R, Odelberg S (University of Utah); Ward D (Yale University); Dausset J, Cohen D and Cann (Centre d'Etude du Polymorphisme Humain, CEPH) (1994) A comprehensive human linkage map with centimorgan density. *Science* **265**: 2049–2054

Nau MM, Brooks BJ, Battey J, Sausville E, Gazdar AF, Kirsch IR, McBride OW, Bertness V, Hollis GF and Minna JD (1985) L-myc, a new myc-related gene amplified and expressed in human small cell lung cancer. *Nature* **318**: 69–73

Nilbert M, Rydholm A, Willen H, Mitelman F and Mandahl N (1994) MDM2 gene amplification correlates with ring chromosomes in soft tissue tumors. *Genes Chrom Cancer* **9**: 261–265

Nilbert M, Rydholm A, Mitelman F, Meltzer PS and Mandahl N (1995) Characterization of the 12q13–15 amplicon in soft tissue sarcomas. *Cancer Genet Cytogenet* **83**: 32–36

Örndal C, Mandahl N, Rydholm A, Willen H, Brosjo O, Heim S and Mitelman F (1992) Supernumerary ring chromosomes in five bone and soft tissue tumors of low borderline malignancy. *Cancer Genet Cytogenet* **60**: 170–175

Oshimura M, Sonta S-i and Sandberg AA (1976) Trisomy of the long arm of chromosome no. 1 in human leukaemia. *J Natl Cancer Inst* **56**: 183–184

Pedeutour F, Suijkerbuijk RF, Van Gaal J, Van de Klundert W, Coindre J-M, Van Haelst A, Collin F, Huffermann K and Turc-Carel C (1993) Chromosome 12 origin in rings and giant markers in well-differentiated liposarcoma. *Cancer Genet Cytogenet* **66**: 133–134

Pedeutour F, Forus A, Coindre J-M, Berner J-M, Nicolo G, Michelis J-F, Terrier P, Ranchere-Vince D, Collin F, Myklebost O and Turc-Carel C (1998) Structure of the supernumerary ring and giant rod chromosomes in adipose tissue tumours. *Gen Chrom Cancer* (in press)

Pedeutour F, Suijkerbuijk RF, Forus A, van Gaal J, van de Klundert W, Coindre J-M, Nicolo G, Collin F, van Haelst U, Huffermann K and Turc-Carel C (1994) Complex composition and co-amplification of SAS and MDM2 in ring and giant rod marker chromosomes in well-differentiated liposarcoma. *Genes Chromosom Cancer* **10**: 85–94

Presland RB, Haydock PV, Fleckman P, Nirunsuksiri W and Dale BA (1992) Characterization of the human epidermal profilaggrin gene. Genomic organization and identification of an S-100 like calcium binding domain at the amino terminus. *J Biol Chem* **267**: 23772–23781

Rogne S, Myklebost O, Høyheim B, Olaisen B and Gedde Dahl TJ (1989) The genes for apolipoprotein AII (APOA2) and the Duffy blood group (FY) are linked on chromosome 1 in man. *Genomics* **4**: 169–173

Rowley JD (1977) Mapping of human chromosomal regions related to neoplasia: evidence from chromosome 1 and 17. *Proc Natl Acad Sci USA* **74**: 5729–5733

Schäfer BW and Heizmann CW (1996) The S100 family of EF hand calcium binding proteins: function and pathology. *Trends Biol Sci* **21**: 134–140

Schäfer BW, Wicki R, Engelkamp D, Mattei M-G and Heizmann CW (1995) Isolation of a YAC clone covering a cluster of nine S100 genes on human chromosome 1q21: rationale for a new nomenclature of the S100 calcium-binding protein family. *Genomics* **25**: 638–643

Schajowich F (1993) *Histological Typing of Bone Tumours*. Springer-Verlag: Berlin

Swallow DM, Gendler S, Griffiths B, Corney G, Taylor-Papadimitriou J and Bramwell ME (1987) The human tumour-associated epithelial mucins are coded by an expressed hyper variable gene locus PUM. *Nature* **328**: 82–84

Szymanska J, Tarkkanen M, Wiklund T, Virolainen M, Blomquist C, Asko-Seljavaara S, Tukiainen E, Elomaa I and Knuutila S (1996) Gains and losses of DNA sequences in liposarcomas evaluated by comparative genomic hybridization. *Genes Chrom Cancer* **15**: 89–94

Szymanska J, Virolainen M, Tarkkanen M, Wiklund T, Asko-Seljavaara S, Tukiainen E, Elomaa I, Blomqvist C and Knuutila S (1997) Overrepresentation of 1q21–23 and 12q13–21 in lipoma-like liposarcomas but not in benign lipomas: a comparative genomic hybridization study. *Cancer Genet Cytogenet* **99**: 14–18

Tarkkanen M, Karhu R, Kallioniemi A, Elomaa I, Kivioja AH, Nevalainen J, Bohling T, Karaharju E, Hyytinen E, Knuutila S and Kallioniemi O-P (1995) Gains and losses of DNA sequences in osteosarcomas by comparative genomic hybridization. *Cancer Res* **55**: 1334–1338

Tsarfati I, Hareuveni M, Horev J, Zaretski J, Weiss M, Jeltsch JM, Garnier JM, Lathe R, Keydar I and Wreschner DH (1990) Isolation and characterization of a hyper-variable gene coding for breast-cancer associated antigen. *Gene* **93**: 313–318

Tucci A, Goldberger G, Whitehead AS, Kay RM, Woods DE and Colten HR (1983) Biosynthesis and postsynthetic processing of human C-reactive protein. *J Immunol* **131**: 2416–2419

Weber-Hall S, Anderson J, McManus A, Abe S, Nojima T, Pinkerton R, Pritchard-Jones K and Shipley J (1996) Gains, losses and amplification of genomic material in rhabdomyosarcoma analyzed by comparative genomic hybridization. *Cancer Res* **56**: 3220–3224

Weiss SW (1994) *Histological Typing of Soft Tissue Tumours*. Springer-Verlag: Berlin

Weith A, Brodeur GM, Bruns GA, Matise TC, Mischke D, Nizetic D, Seldin MF, van Roy N and Vance J (1996) Report of the second international workshop on human chromosome 1 mapping 1995. *Cytogenet Cell Genet* **72**: 113–154

Weterman MAJ, Stoopen GM, van Muijen GNP, Kuznick J, Ruiter DJ and Bloemers HPJ (1992) Expression of calcyclin in human melanoma cell lines correlates with metastatic behaviour in nude mice. *Cancer Res* **52**: 1291–1296

Weterman MAJ, Wilbrink M, Dijkhuizen T, van den Berg E and Geurts van Kessel A (1996) Fine mapping of the 1q21 breakpoint of the papillary renal cell carcinoma-associated (X;1) translocation. *Hum Genet* **98**: 16–21

Wolf M, Aaltonen LA, Szymanska J, Tarkkanen M, Blomquist C, Berner J-M, Myklebost O and Knuutila S (1997) Complexity of 12q13–22 amplicon in liposarcoma: microsatellite repeat analysis. *Genes Chrom Cancer* **18**: 66–70

© Cancer Research Campaign 1998 


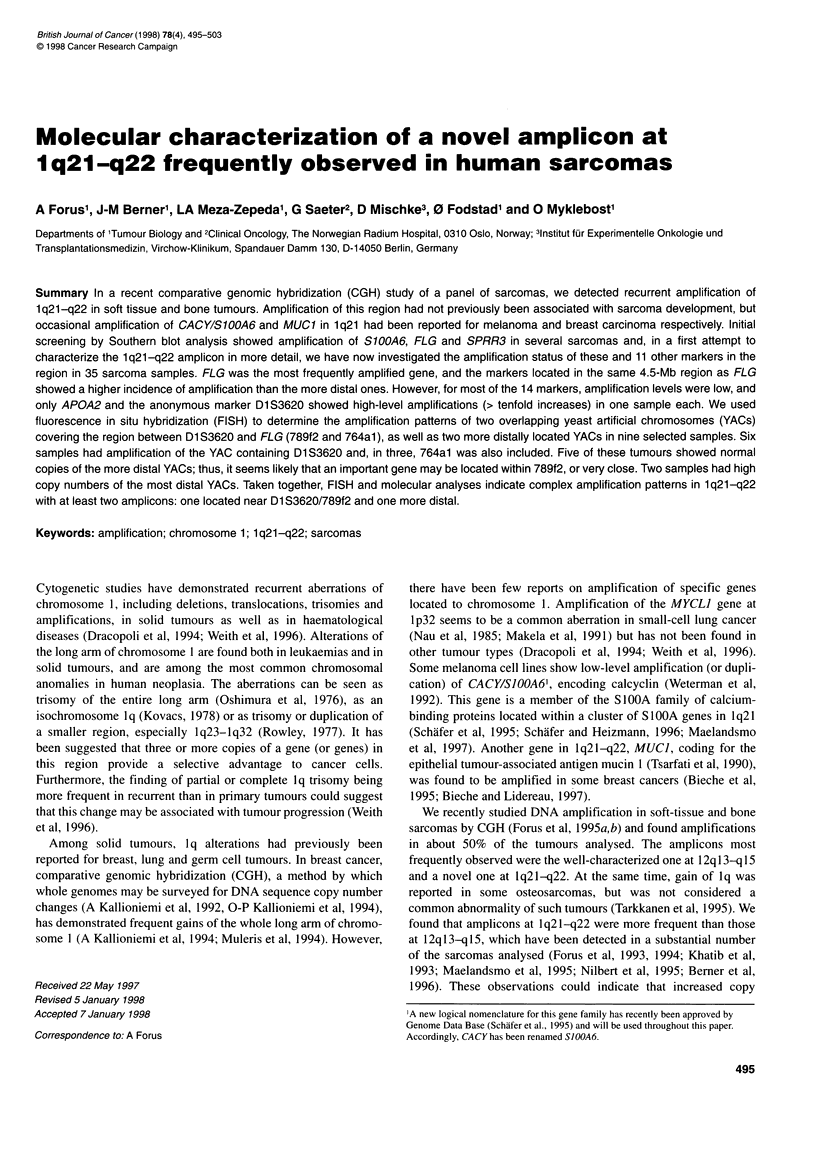

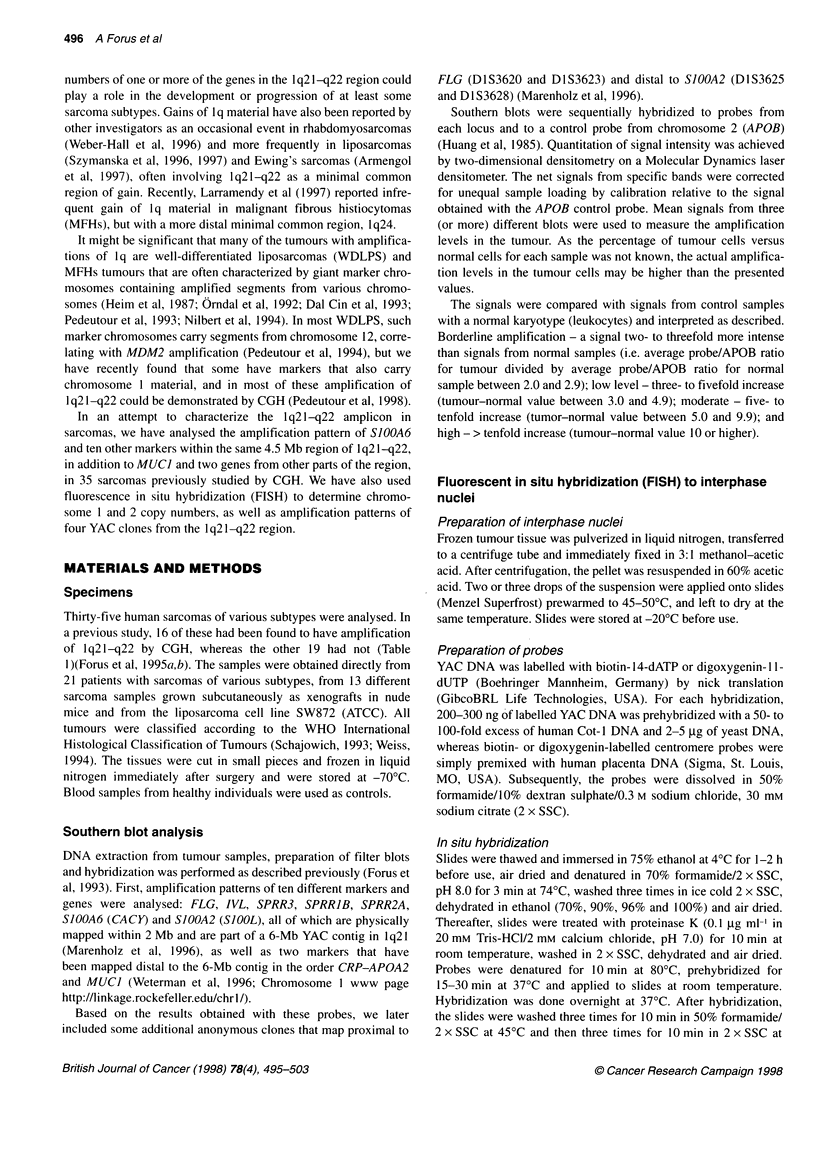

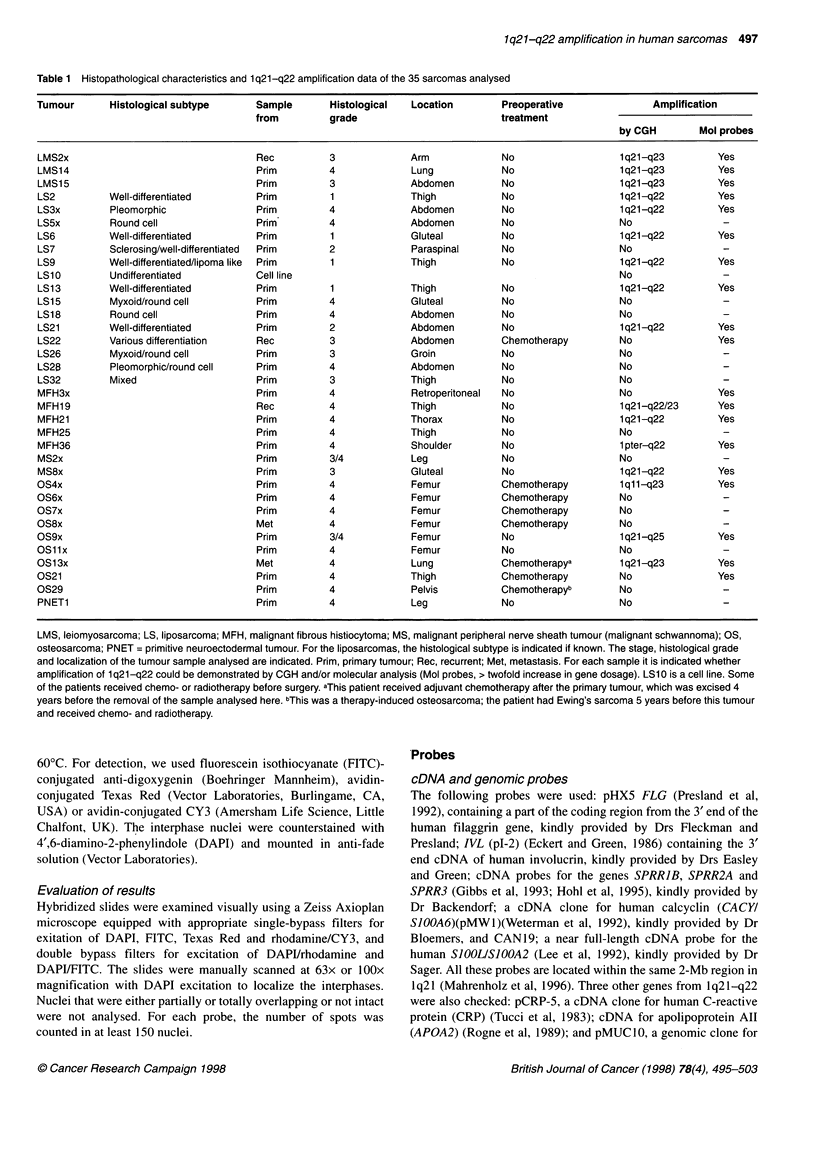

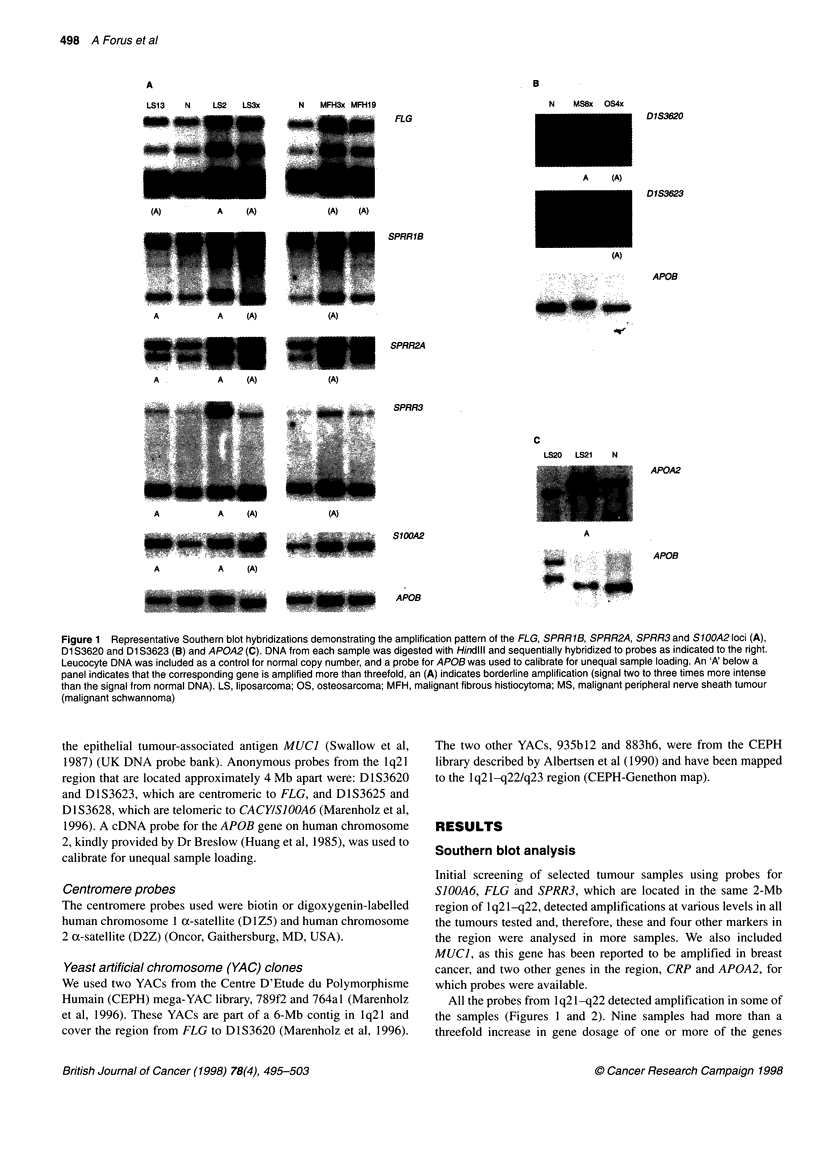

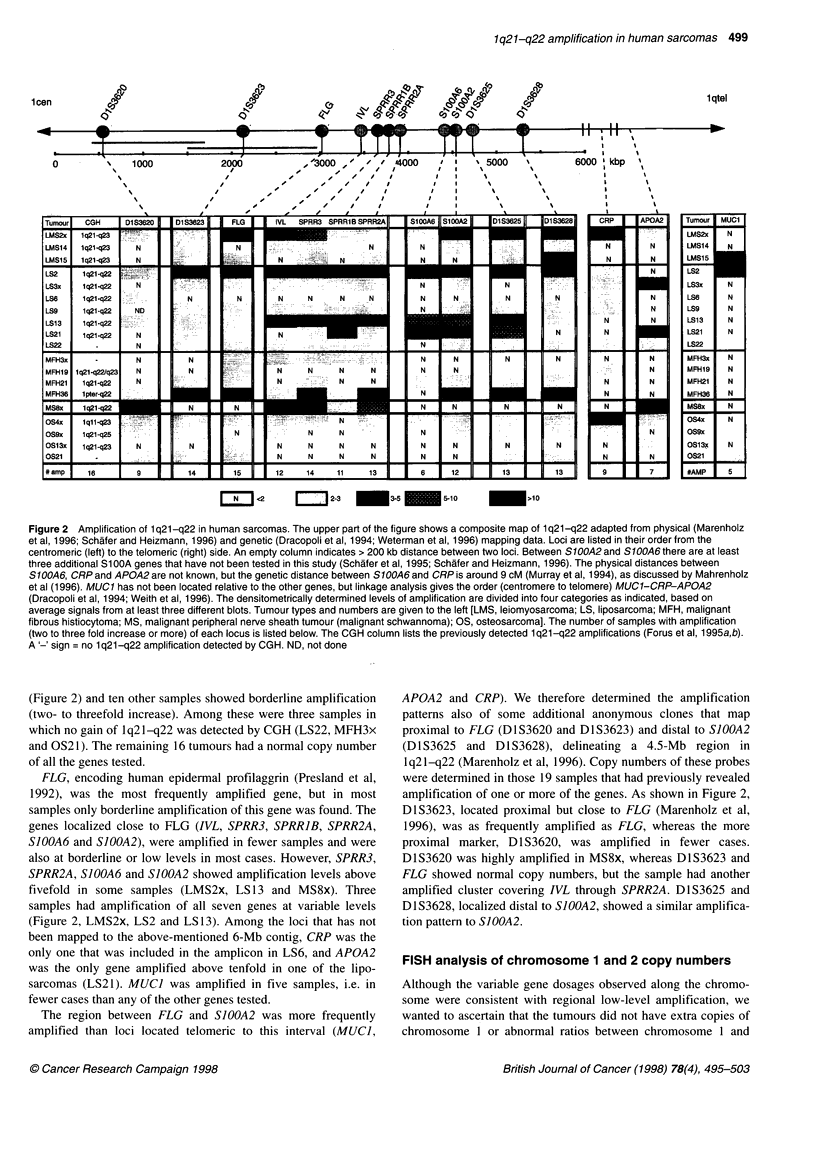

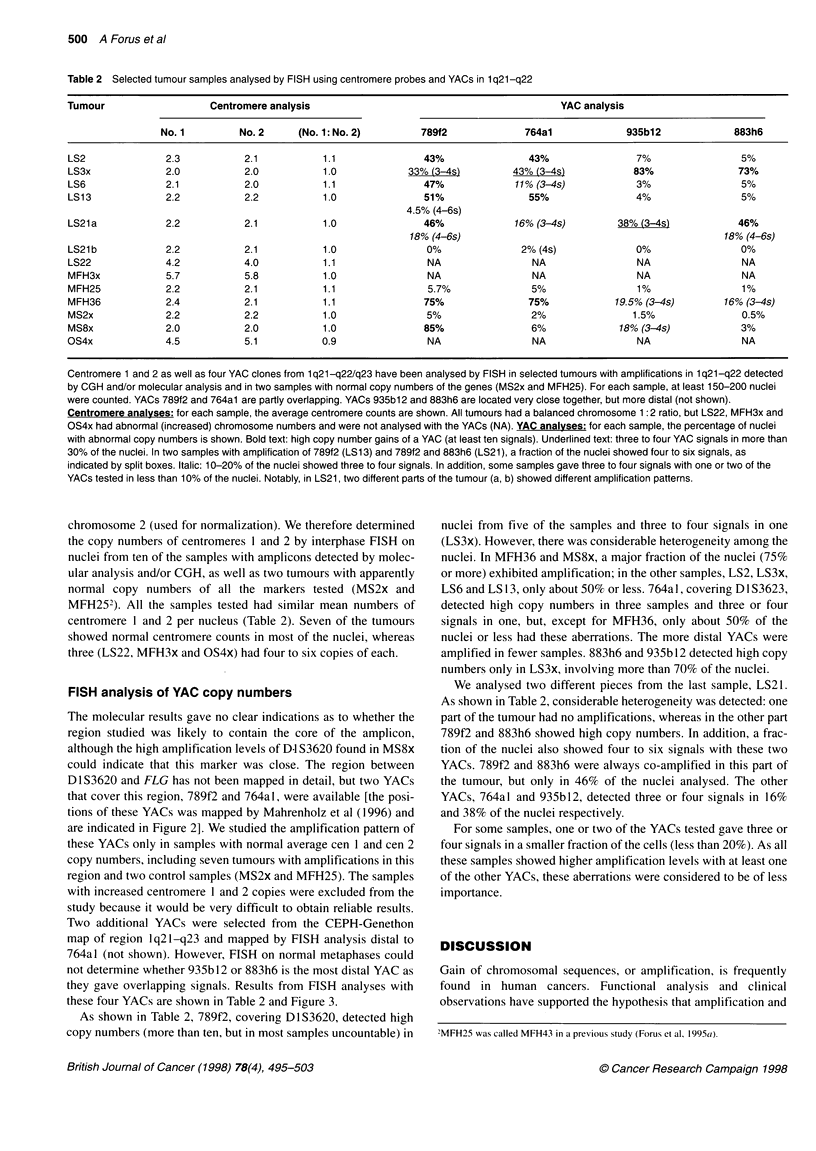

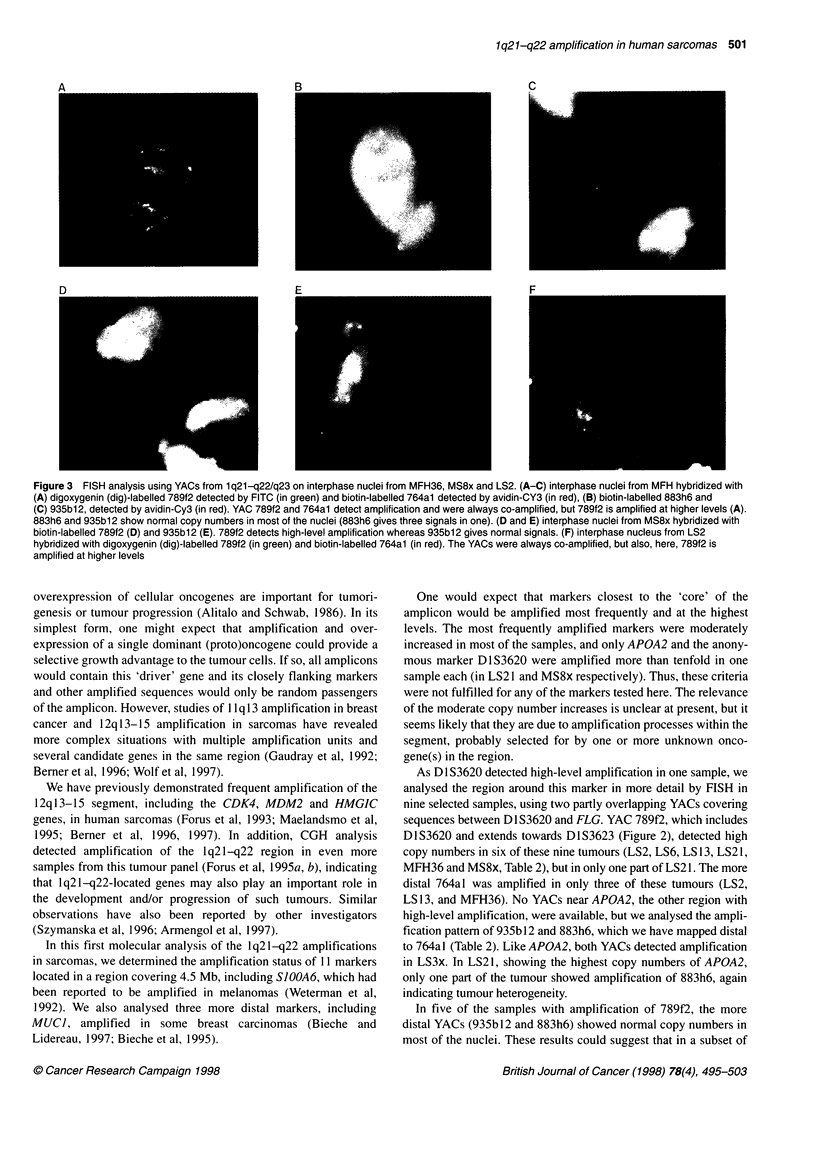

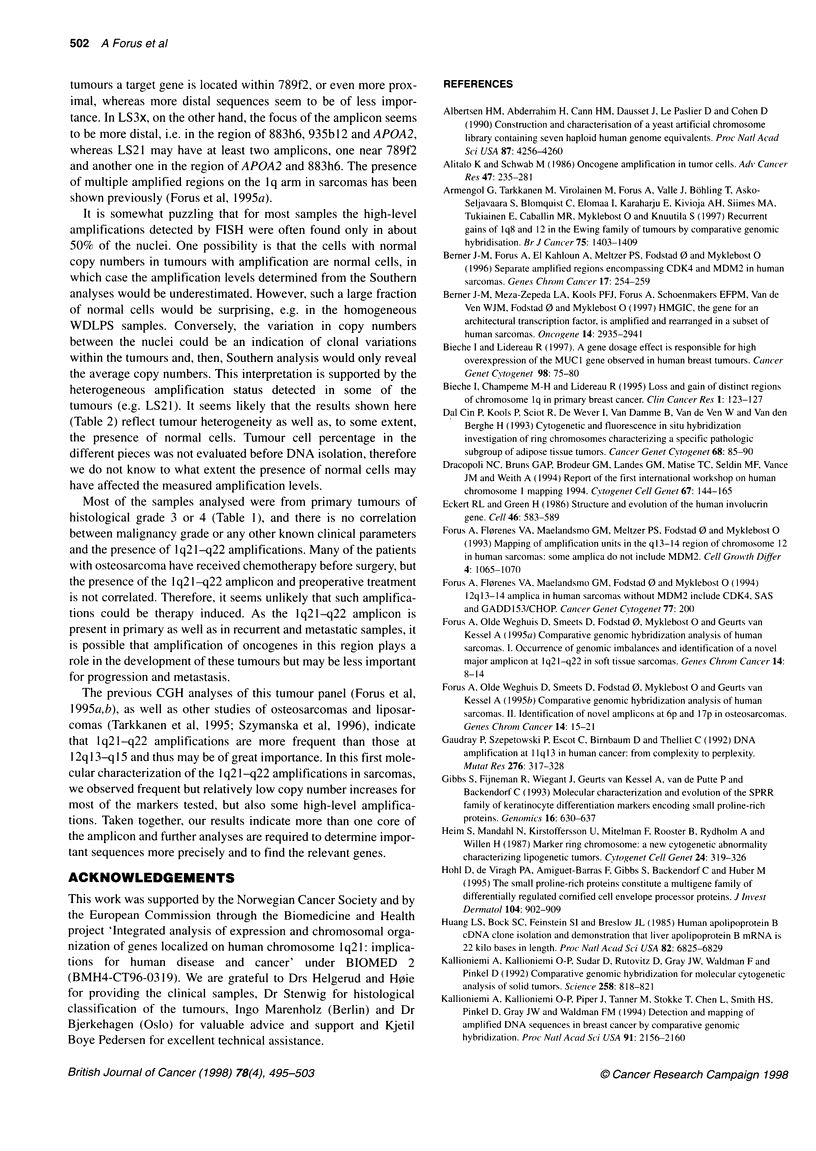

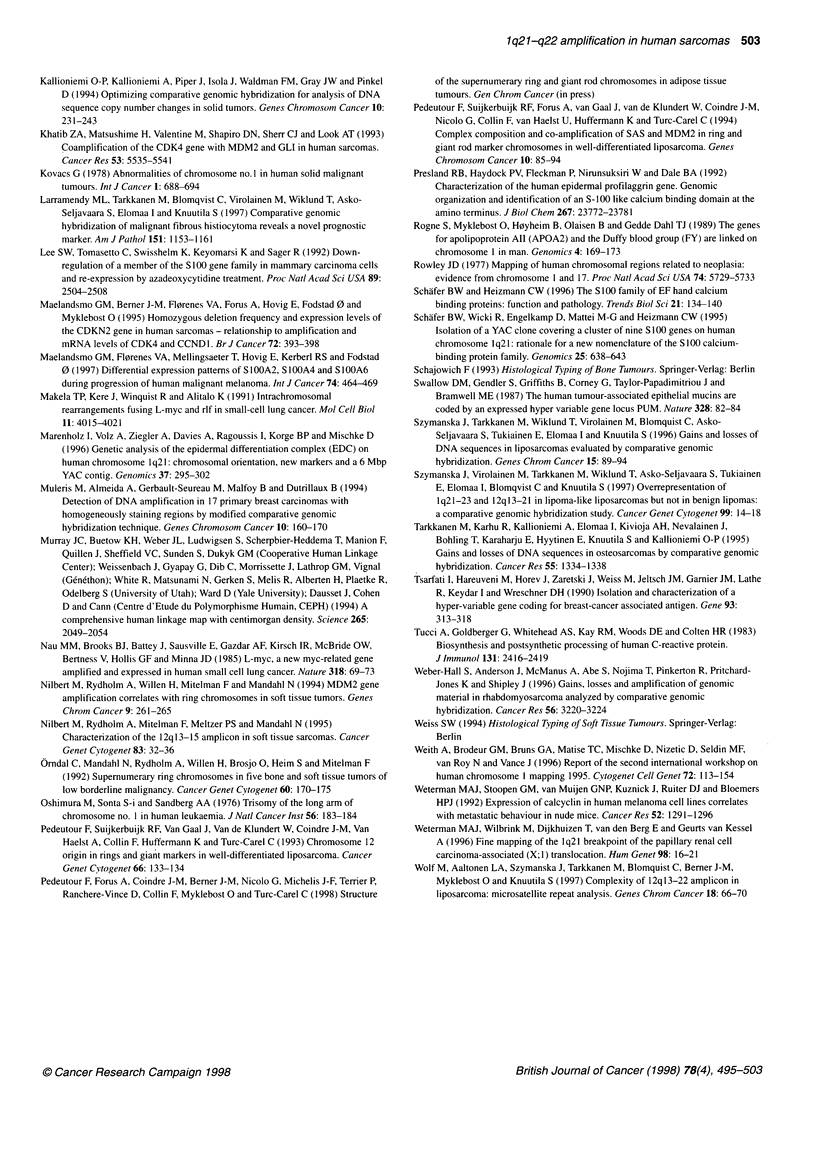


## References

[OCR_01009] Albertsen H. M., Abderrahim H., Cann H. M., Dausset J., Le Paslier D., Cohen D. (1990). Construction and characterization of a yeast artificial chromosome library containing seven haploid human genome equivalents.. Proc Natl Acad Sci U S A.

[OCR_01016] Alitalo K., Schwab M. (1986). Oncogene amplification in tumor cells.. Adv Cancer Res.

[OCR_01022] Armengol G., Tarkkanen M., Virolainen M., Forus A., Valle J., Böhling T., Asko-Seljavaara S., Blomqvist C., Elomaa I., Karaharju E. (1997). Recurrent gains of 1q, 8 and 12 in the Ewing family of tumours by comparative genomic hybridization.. Br J Cancer.

[OCR_01028] Berner J. M., Forus A., Elkahloun A., Meltzer P. S., Fodstad O., Myklebost O. (1996). Separate amplified regions encompassing CDK4 and MDM2 in human sarcomas.. Genes Chromosomes Cancer.

[OCR_01033] Berner J. M., Meza-Zepeda L. A., Kools P. F., Forus A., Schoenmakers E. F., Van de Ven W. J., Fodstad O., Myklebost O. (1997). HMGIC, the gene for an architectural transcription factor, is amplified and rearranged in a subset of human sarcomas.. Oncogene.

[OCR_01045] Bièche I., Champème M. H., Lidereau R. (1995). Loss and gain of distinct regions of chromosome 1q in primary breast cancer.. Clin Cancer Res.

[OCR_01040] Bièche I., Lidereau R. (1997). A gene dosage effect is responsible for high overexpression of the MUC1 gene observed in human breast tumors.. Cancer Genet Cytogenet.

[OCR_01051] Dal Cin P., Kools P., Sciot R., De Wever I., Van Damme B., Van de Ven W., Van den Berghe H. (1993). Cytogenetic and fluorescence in situ hybridization investigation of ring chromosomes characterizing a specific pathologic subgroup of adipose tissue tumors.. Cancer Genet Cytogenet.

[OCR_01056] Dracopoli N. C., Bruns G. A., Brodeur G. M., Landes G. M., Matise T. C., Seldin M. F., Vance J. M., Weith A. (1994). Report and abstracts of the First International Workshop on Human Chromosome 1 Mapping 1994. Bethesda, Maryland, March 25-27, 1994.. Cytogenet Cell Genet.

[OCR_01061] Eckert R. L., Green H. (1986). Structure and evolution of the human involucrin gene.. Cell.

[OCR_01065] Forus A., Flørenes V. A., Maelandsmo G. M., Meltzer P. S., Fodstad O., Myklebost O. (1993). Mapping of amplification units in the q13-14 region of chromosome 12 in human sarcomas: some amplica do not include MDM2.. Cell Growth Differ.

[OCR_01085] Forus A., Weghuis D. O., Smeets D., Fodstad O., Myklebost O., Geurts van Kessel A. (1995). Comparative genomic hybridization analysis of human sarcomas: II. Identification of novel amplicons at 6p and 17p in osteosarcomas.. Genes Chromosomes Cancer.

[OCR_01076] Forus A., Weghuis D. O., Smeets D., Fodstad O., Myklebost O., van Kessel A. G. (1995). Comparative genomic hybridization analysis of human sarcomas: I. Occurrence of genomic imbalances and identification of a novel major amplicon at 1q21-q22 in soft tissue sarcomas.. Genes Chromosomes Cancer.

[OCR_01092] Gaudray P., Szepetowski P., Escot C., Birnbaum D., Theillet C. (1992). DNA amplification at 11q13 in human cancer: from complexity to perplexity.. Mutat Res.

[OCR_01097] Gibbs S., Fijneman R., Wiegant J., van Kessel A. G., van De Putte P., Backendorf C. (1993). Molecular characterization and evolution of the SPRR family of keratinocyte differentiation markers encoding small proline-rich proteins.. Genomics.

[OCR_01103] Heim S., Mandahl N., Kristoffersson U., Mitelman F., Röser B., Rydholm A., Willén H. (1987). Marker ring chromosome--a new cytogenetic abnormality characterizing lipogenic tumors?. Cancer Genet Cytogenet.

[OCR_01108] Hohl D., de Viragh P. A., Amiguet-Barras F., Gibbs S., Backendorf C., Huber M. (1995). The small proline-rich proteins constitute a multigene family of differentially regulated cornified cell envelope precursor proteins.. J Invest Dermatol.

[OCR_01115] Huang L. S., Bock S. C., Feinstein S. I., Breslow J. L. (1985). Human apolipoprotein B cDNA clone isolation and demonstration that liver apolipoprotein B mRNA is 22 kilobases in length.. Proc Natl Acad Sci U S A.

[OCR_01127] Kallioniemi A., Kallioniemi O. P., Piper J., Tanner M., Stokke T., Chen L., Smith H. S., Pinkel D., Gray J. W., Waldman F. M. (1994). Detection and mapping of amplified DNA sequences in breast cancer by comparative genomic hybridization.. Proc Natl Acad Sci U S A.

[OCR_01120] Kallioniemi A., Kallioniemi O. P., Sudar D., Rutovitz D., Gray J. W., Waldman F., Pinkel D. (1992). Comparative genomic hybridization for molecular cytogenetic analysis of solid tumors.. Science.

[OCR_01135] Kallioniemi O. P., Kallioniemi A., Piper J., Isola J., Waldman F. M., Gray J. W., Pinkel D. (1994). Optimizing comparative genomic hybridization for analysis of DNA sequence copy number changes in solid tumors.. Genes Chromosomes Cancer.

[OCR_01141] Khatib Z. A., Matsushime H., Valentine M., Shapiro D. N., Sherr C. J., Look A. T. (1993). Coamplification of the CDK4 gene with MDM2 and GLI in human sarcomas.. Cancer Res.

[OCR_01146] Kovacs G. (1978). Abnormalities of chromosome No. 1 in human solid malignant tumours.. Int J Cancer.

[OCR_01152] Larramendy M. L., Tarkkanen M., Blomqvist C., Virolainen M., Wiklund T., Asko-Seljavaara S., Elomaa I., Knuutila S. (1997). Comparative genomic hybridization of malignant fibrous histiocytoma reveals a novel prognostic marker.. Am J Pathol.

[OCR_01157] Lee S. W., Tomasetto C., Swisshelm K., Keyomarsi K., Sager R. (1992). Down-regulation of a member of the S100 gene family in mammary carcinoma cells and reexpression by azadeoxycytidine treatment.. Proc Natl Acad Sci U S A.

[OCR_01163] Maelandsmo G. M., Berner J. M., Flørenes V. A., Forus A., Hovig E., Fodstad O., Myklebost O. (1995). Homozygous deletion frequency and expression levels of the CDKN2 gene in human sarcomas--relationship to amplification and mRNA levels of CDK4 and CCND1.. Br J Cancer.

[OCR_01169] Maelandsmo G. M., Flørenes V. A., Mellingsaeter T., Hovig E., Kerbel R. S., Fodstad O. (1997). Differential expression patterns of S100A2, S100A4 and S100A6 during progression of human malignant melanoma.. Int J Cancer.

[OCR_01179] Marenholz I., Volz A., Ziegler A., Davies A., Ragoussis I., Korge B. P., Mischke D. (1996). Genetic analysis of the epidermal differentiation complex (EDC) on human chromosome 1q21: chromosomal orientation, new markers, and a 6-Mb YAC contig.. Genomics.

[OCR_01186] Muleris M., Almeida A., Gerbault-Seureau M., Malfoy B., Dutrillaux B. (1994). Detection of DNA amplification in 17 primary breast carcinomas with homogeneously staining regions by a modified comparative genomic hybridization technique.. Genes Chromosomes Cancer.

[OCR_01174] Mäkelä T. P., Kere J., Winqvist R., Alitalo K. (1991). Intrachromosomal rearrangements fusing L-myc and rlf in small-cell lung cancer.. Mol Cell Biol.

[OCR_01203] Nau M. M., Brooks B. J., Battey J., Sausville E., Gazdar A. F., Kirsch I. R., McBride O. W., Bertness V., Hollis G. F., Minna J. D. (1985). L-myc, a new myc-related gene amplified and expressed in human small cell lung cancer.. Nature.

[OCR_01212] Nilbert M., Rydholm A., Mitelman F., Meltzer P. S., Mandahl N. (1995). Characterization of the 12q13-15 amplicon in soft tissue tumors.. Cancer Genet Cytogenet.

[OCR_01207] Nilbert M., Rydholm A., Willén H., Mitelman F., Mandahl N. (1994). MDM2 gene amplification correlates with ring chromosome in soft tissue tumors.. Genes Chromosomes Cancer.

[OCR_01217] Orndal C., Mandahl N., Rydholm A., Willén H., Brosjö O., Heim S., Mitelman F. (1992). Supernumerary ring chromosomes in five bone and soft tissue tumors of low or borderline malignancy.. Cancer Genet Cytogenet.

[OCR_01222] Oshimura M., Sonta S., Sandberg A. A. (1976). Trisomy of the long arm of chromosome No. 1 in human leukemia.. J Natl Cancer Inst.

[OCR_01239] Pedeutour F., Suijkerbuijk R. F., Forus A., Van Gaal J., Van de Klundert W., Coindre J. M., Nicolo G., Collin F., Van Haelst U., Huffermann K. (1994). Complex composition and co-amplification of SAS and MDM2 in ring and giant rod marker chromosomes in well-differentiated liposarcoma.. Genes Chromosomes Cancer.

[OCR_01226] Pedeutour F., Suijkerbuijk R. F., Van Gaal J., Van de Klundert W., Coindre J. M., Van Haelst A., Collin F., Huffermann K., Turc-Carel C. (1993). Chromosome 12 origin in rings and giant markers in well-differentiated liposarcoma.. Cancer Genet Cytogenet.

[OCR_01247] Presland R. B., Haydock P. V., Fleckman P., Nirunsuksiri W., Dale B. A. (1992). Characterization of the human epidermal profilaggrin gene. Genomic organization and identification of an S-100-like calcium binding domain at the amino terminus.. J Biol Chem.

[OCR_01254] Rogne S., Myklebost O., Høyheim B., Olaisen B., Gedde-Dahl T. (1989). The genes for apolipoprotein all (APOA2) and the Duffy blood group (FY) are linked on chromosome 1 in man.. Genomics.

[OCR_01259] Rowley J. D. (1977). Mapping of human chromosomal regions related to neoplasia: evidence from chromosomes 1 and 17.. Proc Natl Acad Sci U S A.

[OCR_01262] Schäfer B. W., Heizmann C. W. (1996). The S100 family of EF-hand calcium-binding proteins: functions and pathology.. Trends Biochem Sci.

[OCR_01266] Schäfer B. W., Wicki R., Engelkamp D., Mattei M. G., Heizmann C. W. (1995). Isolation of a YAC clone covering a cluster of nine S100 genes on human chromosome 1q21: rationale for a new nomenclature of the S100 calcium-binding protein family.. Genomics.

[OCR_01273] Swallow D. M., Gendler S., Griffiths B., Corney G., Taylor-Papadimitriou J., Bramwell M. E. (1987). The human tumour-associated epithelial mucins are coded by an expressed hypervariable gene locus PUM.. Nature.

[OCR_01279] Szymanska J., Tarkkanen M., Wiklund T., Virolainen M., Blomqvist C., Asko-Seljavaara S., Tukiainen E., Elomaa I., Knuutila S. (1996). Gains and losses of DNA sequences in liposarcomas evaluated by comparative genomic hybridization.. Genes Chromosomes Cancer.

[OCR_01283] Szymanska J., Virolainen M., Tarkkanen M., Wiklund T., Asko-Seljavaara S., Tukiainen E., Elomaa I., Blomqvist C., Knuutila S. (1997). Overrepresentation of 1q21-23 and 12q13-21 in lipoma-like liposarcomas but not in benign lipomas: a comparative genomic hybridization study.. Cancer Genet Cytogenet.

[OCR_01290] Tarkkanen M., Karhu R., Kallioniemi A., Elomaa I., Kivioja A. H., Nevalainen J., Böhling T., Karaharju E., Hyytinen E., Knuutila S. (1995). Gains and losses of DNA sequences in osteosarcomas by comparative genomic hybridization.. Cancer Res.

[OCR_01297] Tsarfaty I., Hareuveni M., Horev J., Zaretsky J., Weiss M., Jeltsch J. M., Garnier J. M., Lathe R., Keydar I., Wreschner D. H. (1990). Isolation and characterization of an expressed hypervariable gene coding for a breast-cancer-associated antigen.. Gene.

[OCR_01303] Tucci A., Goldberger G., Whitehead A. S., Kay R. M., Woods D. E., Colten H. R. (1983). Biosynthesis and postsynthetic processing of human C-reactive protein.. J Immunol.

[OCR_01310] Weber-Hall S., Anderson J., McManus A., Abe S., Nojima T., Pinkerton R., Pritchard-Jones K., Shipley J. (1996). Gains, losses, and amplification of genomic material in rhabdomyosarcoma analyzed by comparative genomic hybridization.. Cancer Res.

[OCR_01323] Weterman M. A., Stoopen G. M., van Muijen G. N., Kuznicki J., Ruiter D. J., Bloemers H. P. (1992). Expression of calcyclin in human melanoma cell lines correlates with metastatic behavior in nude mice.. Cancer Res.

[OCR_01328] Weterman M. A., Wilbrink M., Dijkhuizen T., van den Berg E., Geurts van Kessel A. (1996). Fine mapping of the 1q21 breakpoint of the papillary renal cell carcinoma-associated (X;1) translocation.. Hum Genet.

[OCR_01333] Wolf M., Aaltonen L. A., Szymanska J., Tarkkanen M., Blomqvist C., Berner J. M., Myklebost O., Knuutila S. (1997). Complexity of 12q13-22 amplicon in liposarcoma: microsatellite repeat analysis.. Genes Chromosomes Cancer.

